# X-ray reflectivity study of the heat shock protein Hsp70 interaction with an artificial cell membrane model

**DOI:** 10.1038/s41598-023-46066-3

**Published:** 2023-11-06

**Authors:** Ali Makky, Julian Czajor, Oleg Konovalov, Alexander Zhakhov, Alexander Ischenko, Ankita Behl, Shailja Singh, Wasim Abuillan, Maxim Shevtsov

**Affiliations:** 1 Université Paris-Saclay, CNRS, Institut Galien Paris-Saclay, 91400 Orsay, France; 2 Physical Chemistry of Biosystems, Institute of Physical Chemistry, University of Heidelberg, 69120 Heidelberg, Germany; 3 European Synchrotron Radiation Facility (ESRF), 38043 Grenoble, France; 4 Saint-Petersburg Pasteur Institute, Mira Str. 14, 197101 St. Petersburg, Russia; 5 Special Centre for Molecular Medicine, Jawaharlal Nehru University, New Delhi, 110067 India; 6 Klinikum Rechts Der Isar, Technical University of Munich, Ismaninger Str. 22, 81675 Munich, Germany; 7 Institute of Cytology of the Russian Academy of Sciences (RAS), Tikhoretsky Ave. 4, 194064 St. Petersburg, Russia; 8 Personalized Medicine Centre, Almazov National Medical Research Centre, Akkuratova Str. 2, 197341 St. Petersburg, Russia

**Keywords:** Biochemistry, Cancer, Cell biology, Chemical biology

## Abstract

Membrane-bound heat shock protein 70 (Hsp70) apart from its intracellular localization was shown to be specifically expressed on the plasma membrane surface of tumor but not normal cells. Although the association of Hsp70 with lipid membranes is well documented the exact mechanisms for chaperone membrane anchoring have not been fully elucidated. Herein, we addressed the question of how Hsp70 interacts with negatively charged phospholipids in artificial lipid compositions employing the X-ray reflectivity (XRR) studies. In a first step, the interactions between dioleoylphosphatidylcholine (DOPC) in the presence or absence of dioleoylphosphatidylserine (DOPS) and Hsp70 had been assessed using Quartz crystal microbalance measurements, suggesting that Hsp70 adsorbs to the surface of DOPC/DOPS bilayer. Atomic force microscopy (AFM) imaging demonstrated that the presence of DOPS is required for stabilization of the lipid bilayer. The interaction of Hsp70 with DOPC/DOPS lipid compositions was further quantitatively determined by high energy X-ray reflectivity. A systematic characterization of the chaperone-lipid membrane interactions by various techniques revealed that artificial membranes can be stabilized by the electrostatic interaction of anionic DOPS lipids with Hsp70.

The major stress-inducible, ubiquitously expressed 70 kDa heat shock protein (Hsp70, also termed HSPA1A) is highly conserved among different species. As a molecular chaperone Hsp70 plays an important role in processes of protein folding/unfolding, aggregation/disaggregation, as well as degradation (i.e., protein homeostasis)^1,2^. Compared to normal cells, tumor cells have elevated Hsp70 concentrations that contribute to tumorigenesis, invasion and metastasis, as well as resistance to the applied radio-chemotherapies^3,4^. Apart from its cytosolic localization, a plasma membrane-bound Hsp70 expression has been described for tumor but not normal cells^5,6^. Membrane-bound Hsp70 serves as a recognition structure for the natural killer cells and thereby serves as a tumor-specific target for diagnostic and therapeutic approaches^7,8^. Up-to-date several Hsp70-targeting tools (e.g., tumor penetrating peptide (TPP), monoclonal antibody cmHsp70.1 and its Fab fragment, serine protease granzyme B, anticalins) have been successfully employed for tumor imaging and therapy in preclinical models^9–14^. Furthermore, membrane-bound chaperone was also shown to be involved in formation of ion conductance channels^15^, membrane stabilization^16^, signal transduction^17^, clathrin-independent endocytosis^18^, and autophagy^19^. Thus, small heat shock protein HSPB1, was shown to exert a high affinity towards highly fluid membranes that resulted in changes of lateral and rotational lipid mobility of membranes^20^.

The plasma membrane Hsp70 localization could be explained by an association of the chaperone with phosphatidylserine (PS) lipids under stress conditions^21^ or with globoyltriaosylceramide (Gb3) a glycosphingolipid that is present in lipid rafts under non-stress conditions^22^. Subsequent immunocytochemical studies further proved an interaction of Hsp70 chaperone with PS and Gb3 in artificial lipid systems^23,24^. In the plasma membrane heat shock proteins are remarkably flexible macromolecules undergoing conformational changes which are related to the binding and hydrolysis of ATP during its conformational cycle. This interaction appears to be dependent on the presence of phosphatidylserine (PS)^5^.

Although several studies confirmed the incorporation of Hsp70 into the lipid membranes the exact mechanisms of protein-lipid interaction were not fully elucidated. Therefore, the primary aim of this study was to gain insights into the structures and adsorption mechanism of Hsp70 onto artificial lipid bilayer deposited at the solid/liquid interface employing for the first time the high-energy specular X-ray reflectivity (XRR) studies that can provide an insight into the model lipid membrane interacting with a protein^25^. Herein, we combine different physical techniques to analyze the impact of Hsp70 on the structure of cell membrane, quartz crystal microbalance measurements to quantitatively determine the amount of Hsp70 adsorption into the cell membrane and atomic force microscopy to reveal the adsorption mechanisms. The experimental combination between real space and reciprocal space techniques provides a unique tool to unravel interactions of membrane-bound proteins with lipid membrane components.

## Results

### Hsp70 and phospholipid bilayer interaction monitored by QCM-D

**Figure 1:**
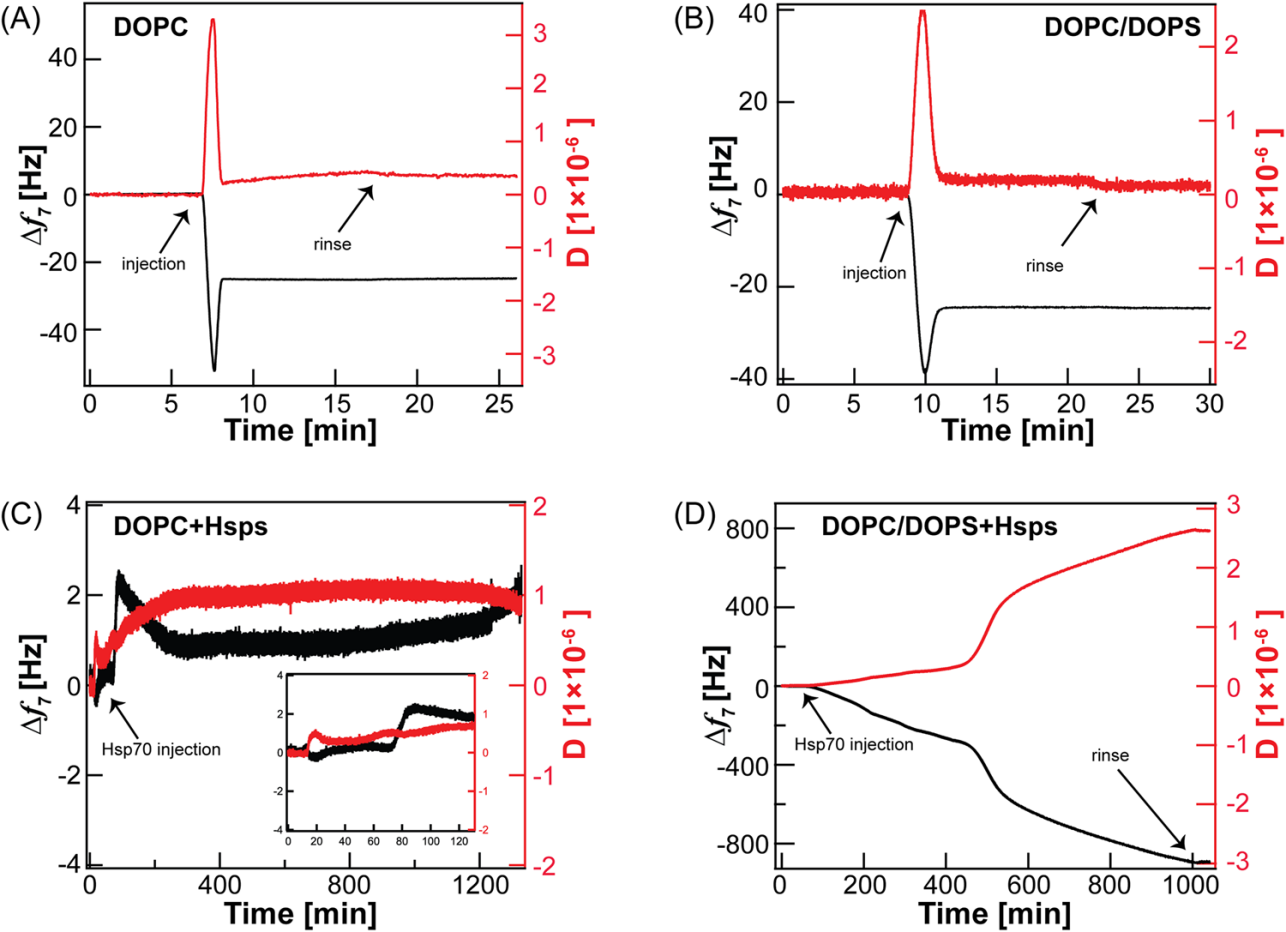
QCM-D experiments showing the changes in resonance frequency (black) and dissipation energy (red) monitored at the frequency mode, n = 7 with *f* = 35 MHz of the formation of (**A**) DOPC bilayer and (**B**) DOPC doped with 20 mol% DOPS bilayer. (**C**) and (**D**) represent the corresponding curves in (**A**) and (**B**) following the injection of Hsp70 in HEPES buffer, respectively. The inset in (**C**) presents the first two hours after the injection of Hsp70.

In order to investigate the adsorption of Hsp70 molecules into phospholipid bilayers, we monitored their kinetics in real time by QCM-D. Figure 1A,B represents the changes in the resonant frequency ∆*f*_*7*_ (black) and in energy dissipation (D) following the injection of DOPC or DOPC/20 mol% DOPS vesicle suspension onto the quartz-SiO_2_ substrate, respectively. A frequency shift of ∆*f*_*7*_ =  − 38.9 Hz and a dissipation of D_7_ = 2.5 × 10^–6^ are observed right after the DOPC/DOPS vesicles injection and then relaxed to ∆*f*_*7*_ =  − 24.5 Hz and D_7_ = 0.2 × 10^–6^. The former frequency shift is due to the adsorption of lipid vesicles onto the substrate and the relaxation is attributed to vesicle fusion which leads to bilayer formation on the substrate. The global shape of frequency and dissipation curves is in a very good agreement to previous reports studied lipid bilayer formation^26–28^.

**Figure 2:**
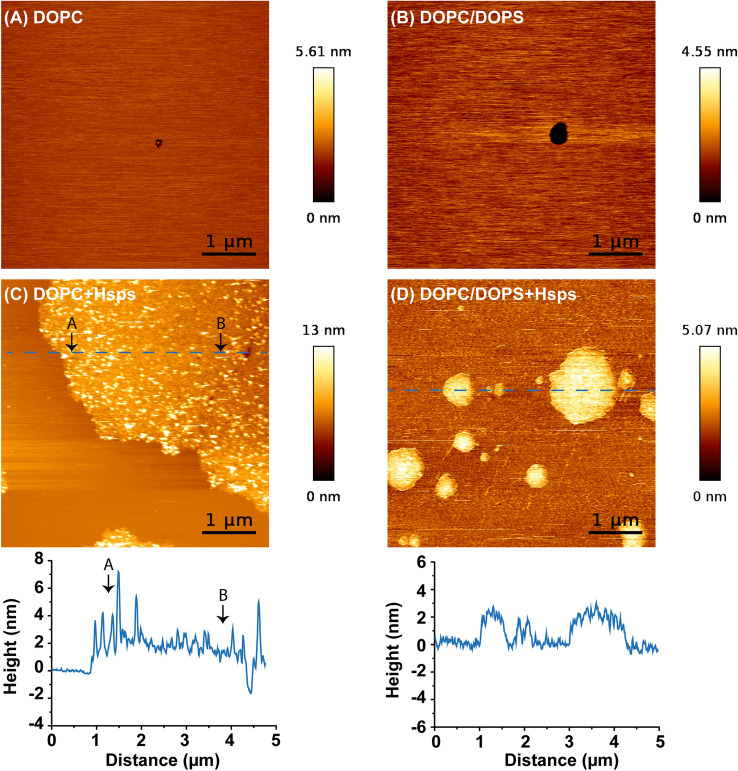
AM-AFM topography images in HEPES buffer (10 mM, NaCl 150 mM, CaCl_2_ 1mM) and room temperature of (**A**) DOPC bilayer and (**B**) DOPC doped with 20 mol% DOPS deposited on mica substrate. The corresponding AFM images after 3 h of incubation Hsp70 at a concentration of 100 µg/ml are shown in (**C**) and (**D**) in the upper panels while the height profiles taken along the dashed lines are presented in the lower panels.

Figure 1C shows the frequency (black) and dissipation (red) changes of DOPC bilayer after the injection of Hsp70 solution (t = 12 min, indicated by arrow). Following the Hsp70 injection, the frequency shifts slightly to a negative value indicating the adsorption of Hsp70 molecules into the DOPC bilayer. However, the frequency shift remains around zero until t = 73 min and then becomes slightly positive and prevails almost constant for more than 20 h. This suggests a mass loss from the substrate surface only within the first hour which can be explained either by a transient removal of the DOPC bilayer due to the surface activity of Hsp70 or by a continuous removal of the DOPC bilayer combined with an adsorption of Hsp70 molecules directly into the substrate. Conversely, the injection of Hsp70 onto the DOPC/DOPS lipid bilayer (Fig. 2D), led to a progressive decrease in ∆*f* which reaches an intensive frequency shift of ∆*f*_7_ ~  − 800 Hz with subsequent increase in the corresponding D_7_ value of ~ 260 × 10^–6^ after 15 h. In addition, both ∆f_7_ and D_7_ remain constant after rinsing with HEPES buffer (arrow, Fig. 1D). This indicates the formation of stable film of Hsp70 proteins onto DOPC/DOPS bilayer.

To further understand the mode of interaction between Hsp70 and lipid bilayer containing DOPS, the normalized changes in frequency (∆*f*) and dissipation (∆D) for the three overtones (n = 3, 5, 7) were plotted (Fig. S1). As shown in this figure, no overlap between the overtones could be observed, thus indicating that the adsorbed Hsp70 molecules form a viscous layer onto the DOPC/DOPS bilayer or significantly alter the mechanical characteristics of the lipid bilayer.

### DOPS is necessary to stabilize lipid bilayer in the presence of Hsp70

In the next step we used AFM to analyze the lipid bilayer structure following the interaction with the Hsp70 molecules. Figure 2A shows DOPC bilayer deposited on mica substrate. The black hole represents a defect in the membrane which was chosen as reference point to confirm the formation of lipid bilayer on the substrate. The bilayer thickness is found to be 4.5 nm which coincides with the well-known thicknesses for similar lipid types (4 – 4.5 nm) reported previously^29–34^.

As shown in Fig. 2C, after 3 h of Hsp70 injection the DOPC bilayer becomes partially covered with a compact protein layer which exhibits a height of 2.1 nm with the presence of small protein aggregates on its top. This indicates that Hsp70 proteins are adsorbed onto the membrane surface. Interestingly, as shown in the line profile of Fig. 2C (lower panel), the protein layer exhibited a height at the boundaries between DOPC bilayer and the adsorbed Hsp70 (indicated by the arrow A) larger than the region indicated by arrow B. This suggests that Hsp70 is adsorbed at the DOPC bilayer in the first step which results in larger height (~ 4 nm) while the thinner region (below 2 nm as indicated by arrow B) suggests the removal of DOPC molecules by Hsp70 proteins. This progressive adsorption and DOPC removal mechanism is supported by slight frequency shift found by QCM-D (Fig. 2C). Over there, the turnover of the frequency shift from negative to positive values suggested the adsorption and mass loss from the substrate surface, respectively.

Similarly, the formation of DOPC/ 20 mol % DOPS bilayer was confirmed by AFM prior the addition of Hsp70. Here, the bilayer thickness was found to be 4.5 nm (Fig. 2B). Interestingly, after the injection of Hsp70, the DOPC/DOPS bilayer becomes rougher which could be due to the adsorption of individual Hsp70 proteins on the bilayer. Moreover, domains of the protein were observed all over the DOPC/DOPS bilayer exhibiting a thickness of approximately 2.2 nm which is comparable to the size of an Hsp70 protein assuming a globular shape (70 kDa, R_min_ ~ 2.7 nm). This suggests that Hsp70 proteins do not disrupt the bilayer structure but adsorb onto the membrane surface. This is consistent to what we found by QCM-D measurements where the mass increased monotonically indicating the continuous adsorption of Hsp70 proteins into DOPC/DOPS membranes for several hours. This finding indicates that the presence of DOPS within the DOPC matrix is necessary to keep the lipid bilayer intact upon the adsorption of Hsp70 proteins. Additionally, adsorption of Hsp70 was assessed in the system consisting of DOPC/DOPS/Chol at 50/20/30 mol% (Fig. S2). Addition of 30 mol% of cholesterol resulted into a phase separation into immiscible liquid ordered–liquid disordered (Lo-Ld) phases. Interestingly, after the addition of Hsp70 proteins, these latter clustered specifically on the Ld domains that might correspond to the DOPS clusters. In addition, we followed the kinetics within 19 h and the bilayer remained intact without materials loss which supports our findings with DOPC/DOPS bilayer (Fig. S3).

### Structure of Hsp70 adsorbed on DOPC bilayer

**Figure 3:**
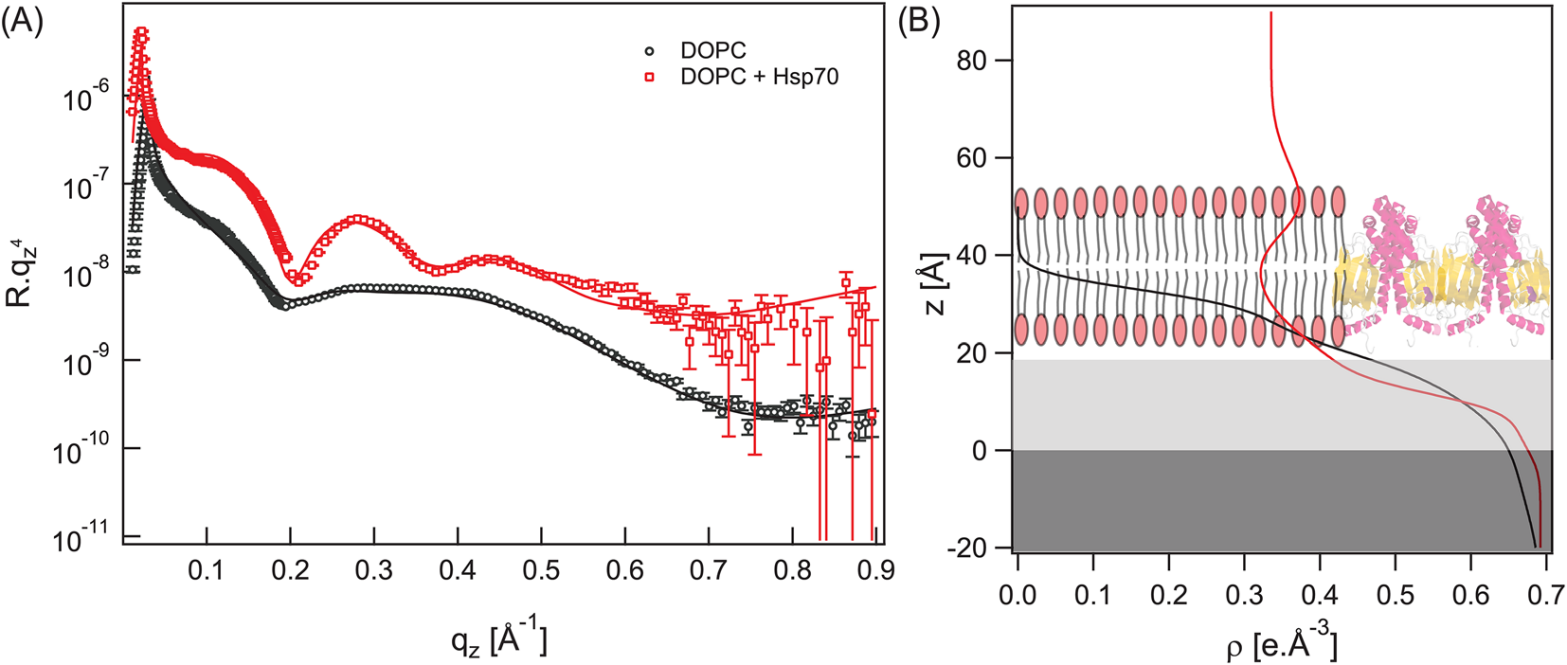
(**A**) X-ray reflectivity curves of the transferred DOPC monolayer in air before (black circles) and after injecting DOPC-Hsp70 proteoliposomes (red squares) together with their best matching fit (solid lines). (**B**) The reconstructed electron density profiles from the best matching fits.

Figure 3A shows the X-ray reflectivity curve of DOPC monolayer deposited on Si-substrate (black circles). To extract the structural parameters the experimental data is fitted with a three-slabs model representing hydrocarbon chains, head group and silicon oxide layer. The reconstructed electron density profile from the best fit is presented in Fig. 3B. The results obtained from the best matching fit (Fig. 3A, black line) are summarized in Table 1. These values are in agreement with values for similar lipid bilayers reported in previous reports^35,36^.

**Table 1:** The obtained structural parameters from the best matching fits in Fig. 3A DOPC/Hsp70 proteoliposomes spread on DOPC monolayer.

	DOPC monolayer		
	d (Å)	ρ (e^−^ × Å^−3^)	σ (Å)
Hydrocarbon chains	11.7 ± 0.3	0.318 ± 0.001	3.0 ± 0.1
Head groups	7.4 ± 0.6	0.510 ± 0.02	4.7 ± 0.3

	DOPC + Hsp70		
Outer head group	12.1 ± 2.1	0.394 ± 0.02	4.9 ± 1.3
Hydrocarbon chains	22.5 ± 1.5	0.275 ± 0.03	5.6 ± 1.8
Inner head group	9.1 ± 0.8	0.494 ± 0.02	7.8 ± 2.1

In the second step, Hsp70 proteoliposomes was injected onto the DOPC monolayer. The corresponding experimental X-ray reflectivity curve after about one hour incubation with Hsp70 is shown in Fig. 3 (red squares). Here we used an additional slab to represent the outer head group and Hsp70 proteins in order to fit the experimental XRR curve. An extra slab to account for Hsp70 protein layer on the lipid bilayer did not lead to any improvement in the fitting quality indicating that the Hsp70 proteins are impeded within the lipid bilayer (Fig. 3B). The reconstructed electron density profile is presented in Fig. 3B and their corresponding structural parameters are summarized in Table 1.

At this time point, the structural parameters of Hsp70/DOPC bilayer presented in Table 1 is consistent with the reported values by Miller et al. for DOPC bilayer where the hydrocarbon chains thickness is 23.2 Å, outer head group 10 Å and 8 Å for inner head group^37^. It should be noted that the inner head group exhibits a smaller thickness due to the reduction in motion of the inner leaflet lipid due the electrostatic and steric interactions with the solid support^38^. However, the roughness values found here are larger than those reported in previous studies of similar lipids especially the roughness at the inner head group/silicon oxide interface (σ ~ 7.8 Å) was the most affected parameter^37,39^. This indicates that Hsp70 proteins penetrate the lipid bilayer and affect even the inner head group region. The main reason why the structure remained similar to that of DOPC bilayer is clear by looking at the inset in Fig. 2C where the DOPC bilayer remained almost intact within the first hour incubation with Hsp70.

In order to monitor the structural changes of HsP70 proteins in DOPC bilayer with time XRR curves were recorded at different incubation time (Fig. S4). Over there the electron density of the hydrocarbon chains increased by 0.044 e^−^ × Å^−3^ within 11 h and most of the lipid bilayer structural parameters were altered within the same time interval (Table S1). This structure changes can be clearly seen from the global shape of the reflectivity curves and the electron density profiles (Fig. S4). For instance, the last XRR curve (at 11 h) was possible to fit using one-slab model suggesting that the bilayer structure is mostly disrupted. Additionally, we calculated the hydration of the outer headgroup bilayers in the absence and the presence of Hsp70. The electron density of the outer head group of DOPC bilayer is 0.387 e^−^/Å^3^ which increased to 0.394 e^−^/Å^3^ after 1 h of incubation with Hsp70. First, we estimated the volume fraction of water (${\mathrm{\varphi }}_{\mathrm{w}})$ on the outer headgroup of DOPC before the addition of Hsp 70 using the following equation:

$$\rho_{measured} = \rho_{H} \left( {1 - \varphi_{w} } \right) + \rho_{water} \varphi_{w}$$

That can be written as follows:

$$\varphi_{w} = \frac{{\rho_{H} - \rho_{measured} }}{{\rho_{H} - \rho_{water} }}$$

where φ_w_ is the volume fraction of water, ${\uprho }_{\mathrm{H}}$ is the electron density of unhydrated headgroup (this can be taken from dry monolayer measurements, ${\uprho }_{\mathrm{H}}$=0.510 e^−^/Å^3^, ρ_measured_ is the total electron density of the outer headgroup of DOPC obtained from the XRR experiment (ρ_measured_ = 0.387 e^−^/Å^3^) and ρ_water_ is the electron density of water (ρ_water_ = 0.335 e^-^/Å^3^). This gives ${\mathrm{\varphi }}_{\mathrm{w}}=70.3\mathrm{\%}$. After the addition of Hsp70 the electron density of the outer head group became ρ_measured_ = 0.394 e^−^/Å^3^. This gives ${\mathrm{\varphi }}_{\mathrm{w}}=66.3\mathrm{\%}$ which means that the presence of Hsp70 reduces the hydration of the outer headgroup by 4%. This corresponds to one water molecule per lipid is being displaced by Hsp70 considering that the area per one lipid molecule is ~ 70 Å^2^ and the head group thickness is 11–12 Å (volume of 770–840 Å^3^) and volume of one water molecule is of 29 Å^3^.

In general, the obtained results from XRR suggest that the DOPC bilayer structure is unstable in the presence of Hsp70 proteins due to the dominant hydrophobic interaction.

### Structure of Hsp70 adsorbed onto DOPC bilayer containing 20 mol% DOPS

**Figure 4:**
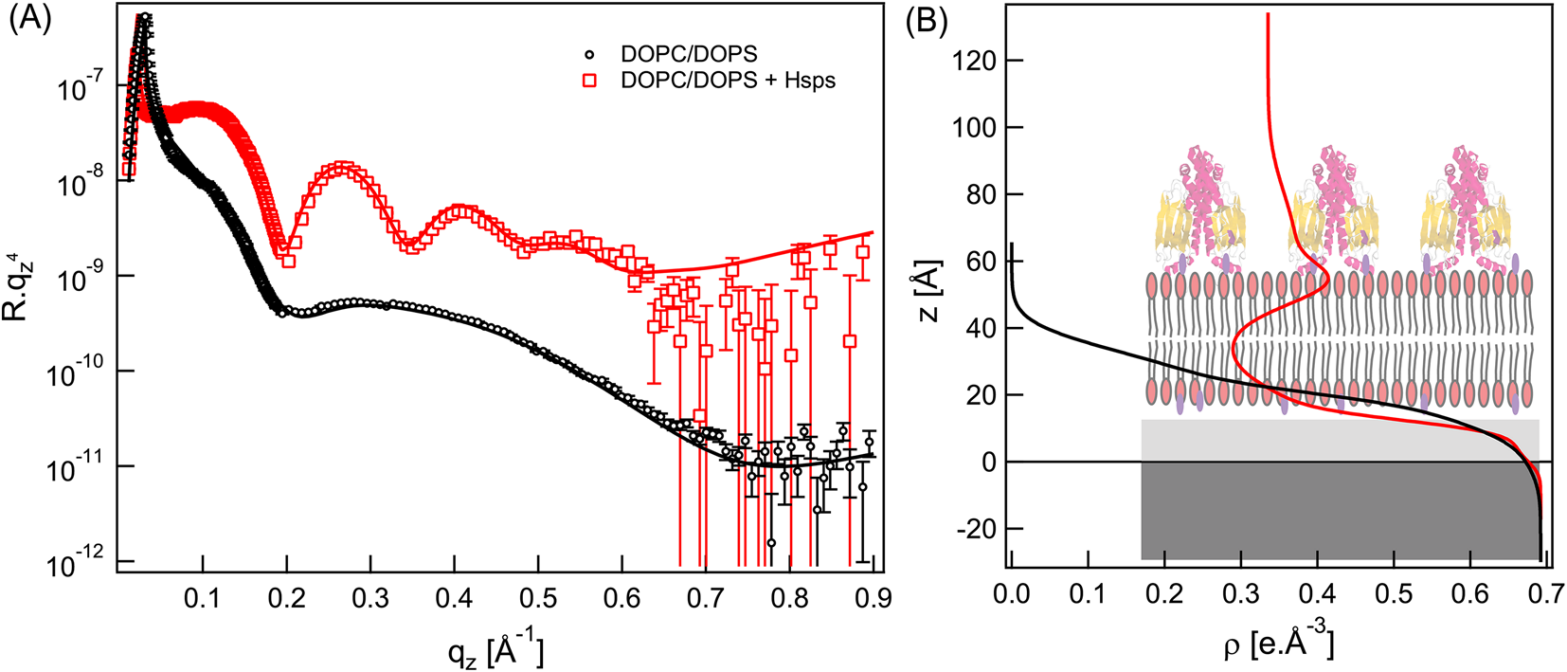
(**A**) Experimental X-ray reflectivity curves of the transferred DOPC/20 mol% DOPS monolayer (black circles) and of DOPC/20 mol% DOPS-HSP70 proteoliposomes (red squares) together with the best matching fits (solid lines). (**B**) The reconstructed electron density profiles from the best matching fits.

Following the last procedure, the monolayer with DOPC doped with 20 mol% DOPS is measured by X-ray reflectivity and fitted with two slabs-model (Fig. 4A, black circles; 4B). After the incubation with proteoliposomes for 1 h XRR curve was recorded (Fig. 4A, red squares; 4B). Here, we used four-slab model with an additional slab to account for Hsp70 protein layer. In fact, from Table 2, it is clear to notice that mainly the electron density of the outer head group have changed compared to that shown in Table S2. This means that Hsp70 protein molecules are adsorbed on the top of DOPC/DOPS bilayer without disturbing the inner lipid bilayer structure.

**Table 2:** The obtained structural parameters of DOPC/DOPS bilayer incorporated with Hsp70 proteins.

	DOPC/DOPS monolayer		
	d (Å)	ρ (e^−^ × Å^−3^)	σ (Å)
Hydrocarbon chains	13.0 ± 0.2	0.248 ± 0.01	5.5 ± 0.3
Head groups	8.0 ± 0.4	0.504 ± 0.008	3.4 ± 0.1

	DOPC/DOPS + Hsp70		
Hsp70 protein layer	22.6 ± 1.5	0.372 ± 0.01	11.9 ± 1.9
Outer head group	10.8 ± 0.6	0.450 ± 0.01	4.7 ± 0.6
Hydrocarbon chains	26.5 ± 0.3	0.284 ± 0.03	6.0 ± 0.6
Inner head group	8.6 ± 0.3	0.406 ± 0.03	6.7 ± 0.6

To monitor the change in structural parameters we recorded XRR curves at different incubation time (Fig. S5). It can be seen from the XRR curves that presence of negatively charged DOPS lipids in the DOPC lipid matrix leads to stable structure upon Hsp70 bounding onto the lipid bilayer. The thickness found here for the protein layer (d = 22.6 Å) clearly indicated that the membrane is covered and/or bound to Hsp70 compared to pure DOPC lipid bilayer. The volume of Hsp70 can be determined from its amino acid sequence with accuracy less than 0.5%^40^. The calculated volume is 84,391 Å^3^ and its size assuming a globular protein shape is estimated to be 5.4 nm in diameter. The lower thickness obtained here for the protein layer is approximately half of the expected value meaning that the DOPC/DOPS bilayer is not fully saturated with Hsp70 proteins. In order to estimate the volume fraction of Hsp70 within the protein layer, the electron density of non-hydrated Hsp70 proteins is estimated from its amino acid sequence to be 0.444 e^−^/Å^3^^41^. Indeed, the measured electron density of the protein layer (*ρ*_*measured*_ = 0.372 e^−^/Å^3^) is in fact an average between water electron density (*ρ*_*water*_ = 0.335 e^−^/Å^3^) and the electron density of Hsp70 proteins (*ρ*_*Hsp*_ = 0.444 e^−^/Å^3^). From these values one can estimate the volume fraction *φ* of Hsp70 proteins as ${\rho }_{measured}={\rho }_{Hsp}\varphi +{\rho }_{water}(1-\varphi )$. The estimated volume fraction is *φ* = 34.3% of Hsp70 proteins on DOPC/DOPS bilayer. Subsequent calculation of the outer head group hydration showed that without Hsp70 ${\mathrm{\varphi }}_{\mathrm{w}}=61.5\mathrm{\%}$ and with Hsp70 it constituted ${\mathrm{\varphi }}_{\mathrm{w}}=31.9\mathrm{\%}$. This indicates that the presence of Hsp70 reduces the hydration of the outer headgroup by 29.6% which corresponds to 8 water molecules per lipid is being displaced by Hsp70. The calculated volume fraction of Hsp70 is smaller than that of bacterial surface proteins bound to lipid monolayers (*φ* > 60%) or to that of lipid anchored proteins (*φ* > 50%)^41,42^. However, this volume fraction seems reasonable owing to the fact that DOPS is 20 mol % in the DOPC lipid matrix. These results suggest that Hsp70 has a higher affinity to DOPS than DOPC lipids. The stable bilayer structure in this case is maintained by the balance between the hydrophobic and electrostatic interactions.

## Discussion

The membrane bound Hsp70 protein found in tumor cells in elevated expression and it is used as a specific target for many imaging diagnostic tools^9,43^. Some previous reports indicated that Hsp70 has a specific interaction with phosphatidylserine containing membranes^21,44^. Indeed, recent studies by Dores-Silva et al. demonstrated the interaction of HSPA5 (Grp78), HSPA8 (HSC70), and HSPA9 (mortalin, mtHsp70) with negatively charged phospholipids (including phosphatidylserine and cardiolipin) that was driven by the increases in entropy and decreased by the presence of ATP or ADP^45–47^. Herein, we designed a model system of solid supported artificial lipid bilayer to unravel the interaction between Hsp70 proteins and lipid bilayer. Quartz crystal microbalance experiments showed minor frequency shift after the addition of Hsp70 proteins on DOPC bilayer which indicated mass loss from the substrate surface. In details, this mass loss is elucidated by AFM measurements where the continuous removal of DOPC bilayer is followed by Hsp70 adsorption onto the substrate surface. Both experiments suggested the unstable DOPC bilayer in the presence of Hsp70. On the contrary, DOPS containing lipid bilayer exhibited a continuous frequency shift of up to -800 Hz suggesting the adsorption of Hsp70 proteins onto the lipid bilayer. These data are in line with previous studies by Lamprecht et al. that demonstrated the insertion of Hsp70 into dipalmitoyl phosphatidylserine (DPPS) domains and formation of clusters with increasing the protein density^24^. The stable bilayer structure upon Hsp70 adsorption is further confirmed by AFM topographic images showing a micrometer sized domains with 4 nm thickness.

To further assess the interaction of Hsp70 with lipids we for the first time performed X-ray reflectivity studies^48^. XRR measurements showed that in the first hour of incubation with Hsp70 proteoliposomes a bilayer containing Hsp70 proteins is formed on the silicon substrate surface. The structural parameters indicated that Hsp70 protein molecules are impeded within the bilayer and in contact with the substrate (Table 1). This bilayer structure could not be maintained longer than 11 h indicating that the interaction between Hsp70 proteins and neutral lipid molecules is mainly governed by hydrophobic interaction. In the presence of DOPS lipids the bilayer structure remained intact for more than 21 h and stable protein layer with an average thickness of 22.6 Å and electron density of 0.372 e^-^/Å^3^ is maintained. The later enabled us to estimate the volume fraction of Hsp70 to be 34.3% pointing towards the anchoring mechanism between Hsp70 and lipid bilayer is mediated the electrostatic interaction between Hsp70 proteins and PS lipids. Of note, membrane adsorption of protein resulted in the changes in hydration of the heads and the tails of a lipid bilayer. Thus, the increase of the electron density of the outer head group of DOPC bilayer by ∆ ρ = 0.007 e^−^/Å^3^ in the presence of Hsp70 leads to a reduction of the headgroup hydration by 4% which corresponds to one displaced water molecule per one lipid molecule. However, Hsp70 induces a significant decrease of the DOPC/DOPS headgroup hydration level by 29.6% which corresponds to the displacement of 8 water molecules per lipid. Our experimental findings provide quantitative evidence that the affinity of Hsp70 to PS is primarily mediated by electrostatic interactions. The underlying mechanism of the accumulation of Hsp70 in lipid bilayer containing PS can be explained by a balance between electrostatic and hydrophobic interactions.

In conclusion, our studies employing XRR measurements confirmed the insertion of the Hsp70 into the lipid bilayer mediated by electrostatic and hydrophobic interactions. These findings along with previous studies employing other methods for analysis of chaperone anchoring with the membrane lipids (e.g., AFM, biochemical methods, etc.) provide the proof of Hsp70 presence in the cell membranes.

## Methods

### Heat shock protein Hsp70

Recombinant human heat shock protein 70 was derived from *E.coli* bacteria transformed with a pMSHSP plasmid and purified using two-step chromatography procedure as described previously^49^. Briefly, Hsp70 was purified employing DEAE-Sepharose (GE Healthcare, Pittsburgh, PA, USA) for anion exchange chromatography with subsequent ATP-affinity chromatography using ATP-Agarose (Sigma-Aldrich Co. LLC., St. Louis, MO, USA). Following depletion of bacterial lipopolysaccharide (LPS) using polymyxin B-agarose gel (Sigma-Aldrich, USA) was lyophilized and stored at  − 20 °C. The endotoxin content was below 0.1 EU/mg Hsp70 (as measured by Limulus Amebocyte Lysate (LAL) assay, Lonza). Additionally, the mass spectrometry (MS) analysis of Hsp70 was performed. MS spectra were searched in-house using the Mascot algorithm (version 2.2.0). Artificial lipid bi- and monolayers were produced with two lipids that possess identical hydrocarbon chains but different head groups: DOPC (1,2-dioleoyl-*sn*-glycero-3-phosphocholine) and DOPS (1,2-dioleoyl-*sn*-glycero-3–phosphoserine) as anionic lipids (Avanti polar lipids, Alabaster, USA). Unless stated otherwise, all other chemicals, which were used without purification, were purchased either from Sigma-Aldrich (Munich, Germany) or from Carl Roth (Karlsruhe, Germany). HEPES buffered saline containing 150 mM NaCl, 10 mM HEPES (4-(2-hydroxyethyl)-1-piperazine ethane sulfonic acid), and 1 mM CaCl_2_ adjusted to pH 7.4 was used throughout all experiments in this study. Deionized water from a Milli-Q device (Millipore, Molsheim, France) was used throughout this study.

### Sample preparation

Vesicle suspensions of DOPC or DOPC doped with 20 mol % DOPS were prepared by tip sonication for 30 min and then centrifuged to remove titanium particles. The final vesicle suspension concentration is 1 mg/ml in HEPES buffer. Lipid monolayers were deposited at constant surface pressure of 20 mN/m using Langmuir film balance (Biolin Scientific, Finland). Hsp70 proteoliposomes were prepared following the protein reconstitution methods described previously^50^. Briefly, Hsp70 lyophilized powder was dissolved in HEPES buffer with a concentration of 2 mg/ml. The protein solution was mixed with lipid suspension (1 mg/ml) containing 2% SDS at a protein to lipid ratio of 1:500. SDS was removed by dialysis overnight in pure HEPES buffer. The proteoliposomes were separated from free Hsp70 proteins by size exclusion column. The fractions were checked by dynamic light scattering to confirm the presence of proteoliposomes (Nano-zetasizer ZS, Malvern Panalytical, UK). The incorporation of Hsp70 proteins into the lipid vesicles is confirmed by the absorbance at 280 nm using UV–visible spectrometer (Thermo-scientific, USA).

### Quartz crystal with dissipation monitoring (QCM-D)

The adsorption kinetics of Hsp70 on lipid bilayers was studied using a QCMD E4 instrument from Q-Sense (Gothenburg, Sweden) equipped with four independent channels and a peristaltic pump. The silicon dioxide (SiO_2_) coated quartz crystals (f_0_ = 5 MHz) were supplied by Q-Sense (Gothenburg, Sweden). Prior to their use, the crystals were soaked in 10 mM sodium dodecyl sulfate (SDS) solution for 1 h, followed by rinsing with ultrapure water, dried under a N_2_ stream, and treated in a UV-ozone chamber for 20 min. Vesicle suspensions (0.2 mM in HEPES) were flowed on the SiO_2_-coated quartz crystals for 15 min until the change in frequency Δf and dissipation D signals were stable. Then, the lipid bilayer was rinsed in HEPES buffer for 10 min. Finally, the solution of Hsp70 (10 µg/ml in HEPES buffer) was injected and monitored for 1 day. The flow rate of the peristaltic pump was set to 100 μL/min, and the temperature was stabilized at 25 ± 0.1 °C.

### Atomic force microscopy imaging (AFM)

For AFM imaging, 100 µL of the vesicle suspensions at 1 mM of DOPC and DOPC doped with 20 mol% DOPS were deposited on freshly cleaved mica (1 cm^2^, muscovite mica, grade V1 from Tedpella) and left for 1 h at room temperature (25 °C) to ensure the formation of lipid bilayer. Afterwards, the mica surfaces were rinsed thoroughly with HEPES buffer to remove the excess of unbroken liposomes. The AFM imaging of lipid bilayer was performed in HEPES buffer at room temperature (25 °C) using a JPK Nanowizard Ultraspeed AFM (Bruker, Karlsruhe, Germany) in amplitude modulation AFM (AM-AFM) with low force settings (80–90% of the free amplitude A ~ 6 nm). Gold coated silicon nitride Lever (SNL-A) probes (Bruker, Santa Barbara, CA) with a nominal spring constant of 0.35 N/m and a tip radius of 2 nm have been used. Afterwards, 50 µl of Hsp70 solution (1 mg/ml) was added onto the lipid bilayer to get a final Hsp70 concentration of 100 µg/ml in the fluid AFM cell and the adsorption mechanism was monitored over approximately 24 h.

AFM images of 5 µm x 5 µm (512 × 512 pixels) were taken at the scan rate of 1 Hz. The height images were processed using the JPK Data Processing software.

### High energy specular X-ray reflectivity (XRR)

**Figure 5:**
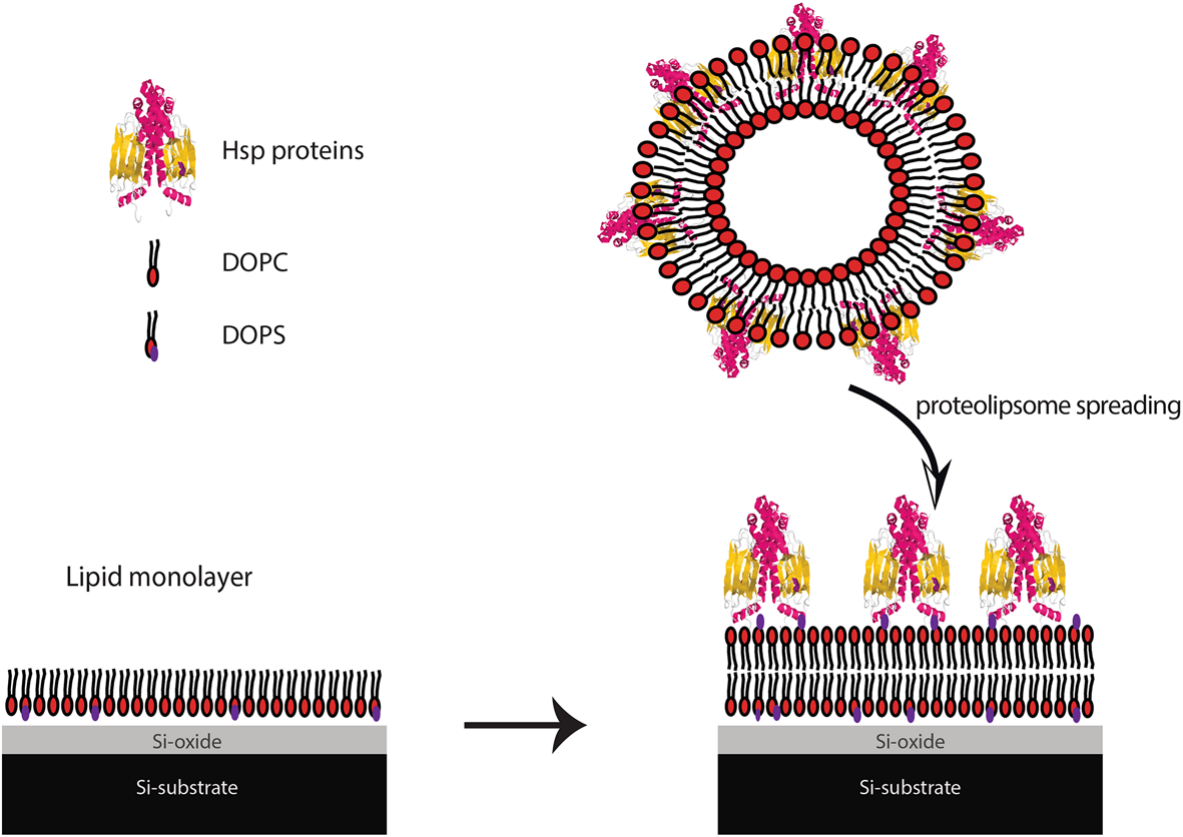
Lipid monolayer were deposited by Langmuir film balance by Langmuir–Blodgett transfer (left panel) followed by the injection of Hsp70-containing proteoliposomes (right panel). The protein structure is adapted from (PDB: ID 1DKZ).

For XRR measurements, the prepared proteoliposomes containing Hsp70 proteins were injected on the top of dry Langmuir monolayer deposited on Si-substrate (Fig. 5). This preparation method used to insure the highest protein to lipid ratio.

XRR experiments were carried out at the beamline ID10 of the European Synchrotron Radiation Facility (ESRF, Grenoble). The samples were irradiated with a monochromatic synchrotron beam with an energy of 22 keV (λ = 0.56 Å). XRR was recorded with a linear position sensitive detector (Mythen 1 K, Dectris, Switzerland) and the XRR curves were obtained by integrating the intensity near the specular plane followed by background subtraction. The reflectivity was normalized to the incident beam and analyzed using the Abeles matrix with a genetic minimization algorithm implemented in the MOTOFIT software package^51^. The incident angle *α*_*i*_ was transformed into the scattering vector component normal to the interface, ${q}_{z}=4\pi \mathrm{sin}({\alpha }_{i})/\lambda$. During the refinement of the reflectivity curves the electron density of silicon, silicon oxide and buffer were set at constant values of 0.692, 0.660 and 0.335e^−^ × Å^−3^, respectively. The silicon oxide layer thickness was found to be between 12 and 25 Å.

### Statistics

The one-way analysis by Kruskal–Wallis was applied for detection of the differences. The software program used for the statistical analysis was Statistica Version 9.2. In all experiments, distinctions were regarded as statistically reliable at *P* < 0.05.

### Supplementary Information

Supplementary Information.

## Data Availability

The datasets used and/or analyzed during the current study available from the corresponding authors (Dr. Wasim Abuillan, Dr. Maxim Shevtsov) on reasonable request.
