# Supplementary material for: X-ray reflectivity study of the heat shock protein Hsp70 interaction with an artificial cell membrane model
Source: Sci Rep. 2023 Nov 6;13:19157. doi: 10.1038/s41598-023-46066-3 (PMC10628213; doi:10.1038/s41598-023-46066-3)
Supplement: Supplementary file 1 — Supplementary Information. [file 41598_2023_46066_MOESM1_ESM.docx]

Supplementary Material

**X-ray reflectivity study of the heat shock protein Hsp70 interaction with an artificial cell membrane model**

Ali Makky^1§^, Julian Czajor^2^, Oleg Konovalov^3^, Alexander Zhakhov^4^, Alexander Ischenko^4^, Ankita Behl^5^, Shailja Singh^5^, Wasim Abuillan^2*§^, Maxim Shevtsov^6–8*§^

^1^Université Paris-Saclay, CNRS, Institut Galien Paris-Saclay, 91400 Orsay, France

^2^Physical Chemistry of Biosystems, Institute of Physical Chemistry, University of Heidelberg, 69120 Heidelberg, Germany

^3^European Synchrotron Radiation Facility (ESRF), 38043 Grenoble, France

^4^Saint-Petersburg Pasteur Institute, Mira Str. 14, 197101 St. Petersburg, Russia

^5^Special Centre for Molecular Medicine, Jawaharlal Nehru University, New Delhi 110067, India

^6^Klinikum rechts der Isar, Technical University of Munich, 81675 Munich, Germany

^7^Institute of Cytology of the Russian Academy of Sciences (RAS), 194064 St. Petersburg, Russia

^8^Personalized Medicine Centre, Almazov National Medical Research Centre, Akkuratova Str. 2,197341 St. Petersburg, Russia

^§^Shared last co-authorship

^*^Corresponding authors: waseemrb@googlemail.com (W. Abuillan), [maxim.shevtsov@tum.de](mailto:maxim.shevtsov@tum.de) (M. Shevtsov).

**Supplementary Figure S1: overlay of the frequency overtones (n = 3, 5, 7) and the corresponding dissipation as a function of time.**


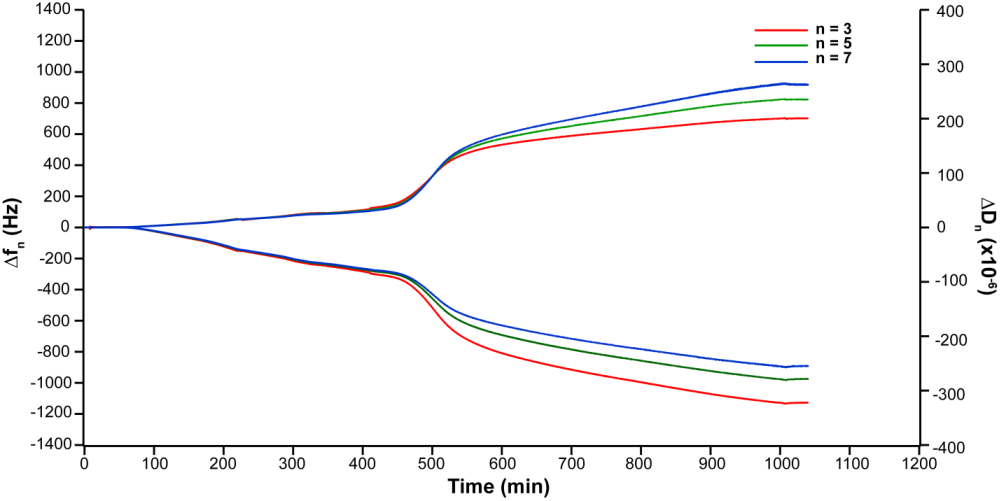


**Fig. S1**: The normalized change in frequency (Δ*f*n) and dissipation (Δ*D*) as a function oftime, recorded for the three overtones (red: *n* = 3, green: *n* = 5, and blue: *n* = 7; *f*_0_ = 5 MHz).

**Supplementary Figure S2: AM-AFM topography images in HEPES buffer at room temperature of DOPC/DOPs/Chol bilayer (50/20/30 mol%) before and after addition of Hsp70.**

*
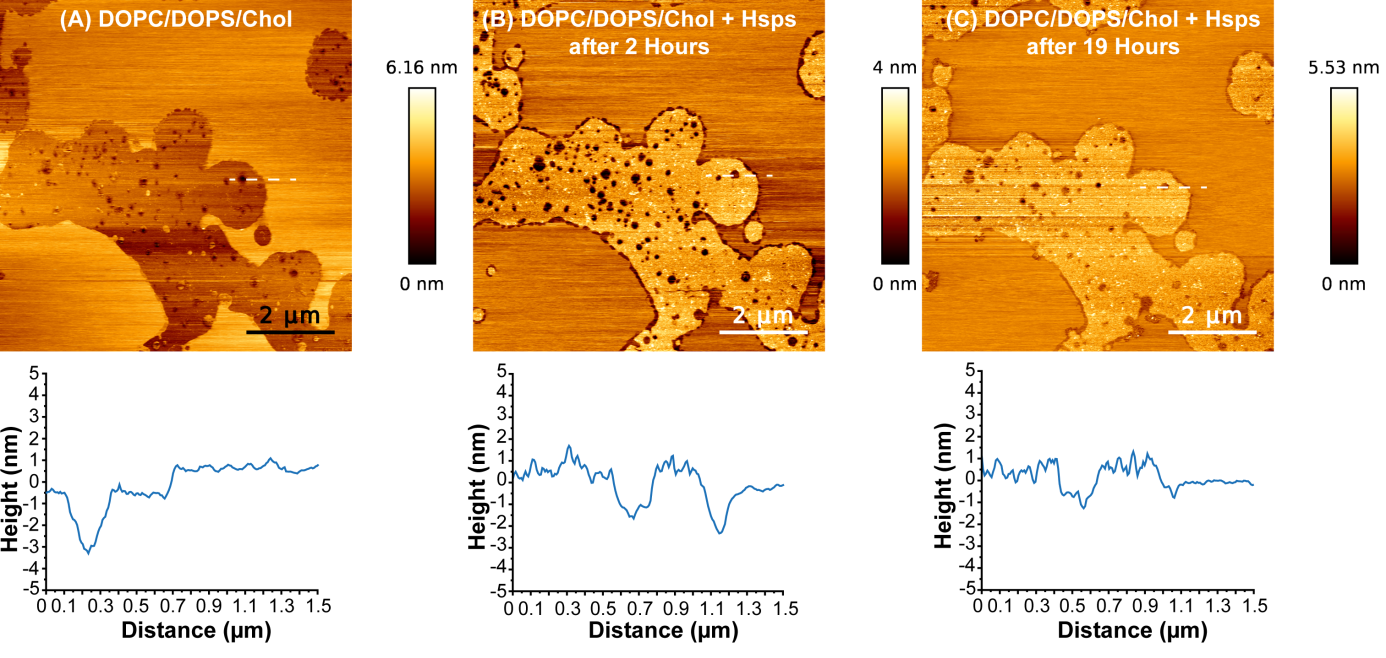
*

**Fig. S2:** AM-AFM topography images in HEPES buffer at room temperature of DOPC/DOPs/Chol bilayer (50/20/30 mol%) deposited on mica substrate before addition of Hsp70 (A) and after Hsp70 addition at a concentration of 100 µg/ml and incubation of 2 hours (B) and 19 hours (C). The height profiles taken along the dashed lines are presented in the lower panels.

**Supplementary Figure S3: AM-AFM topography images in HEPES buffer at room temperature, of DOPC/DOPs/Chol bilayer (50/20/30 mol%) before and after addition of Hsp70 for a period of 19 h.**

*
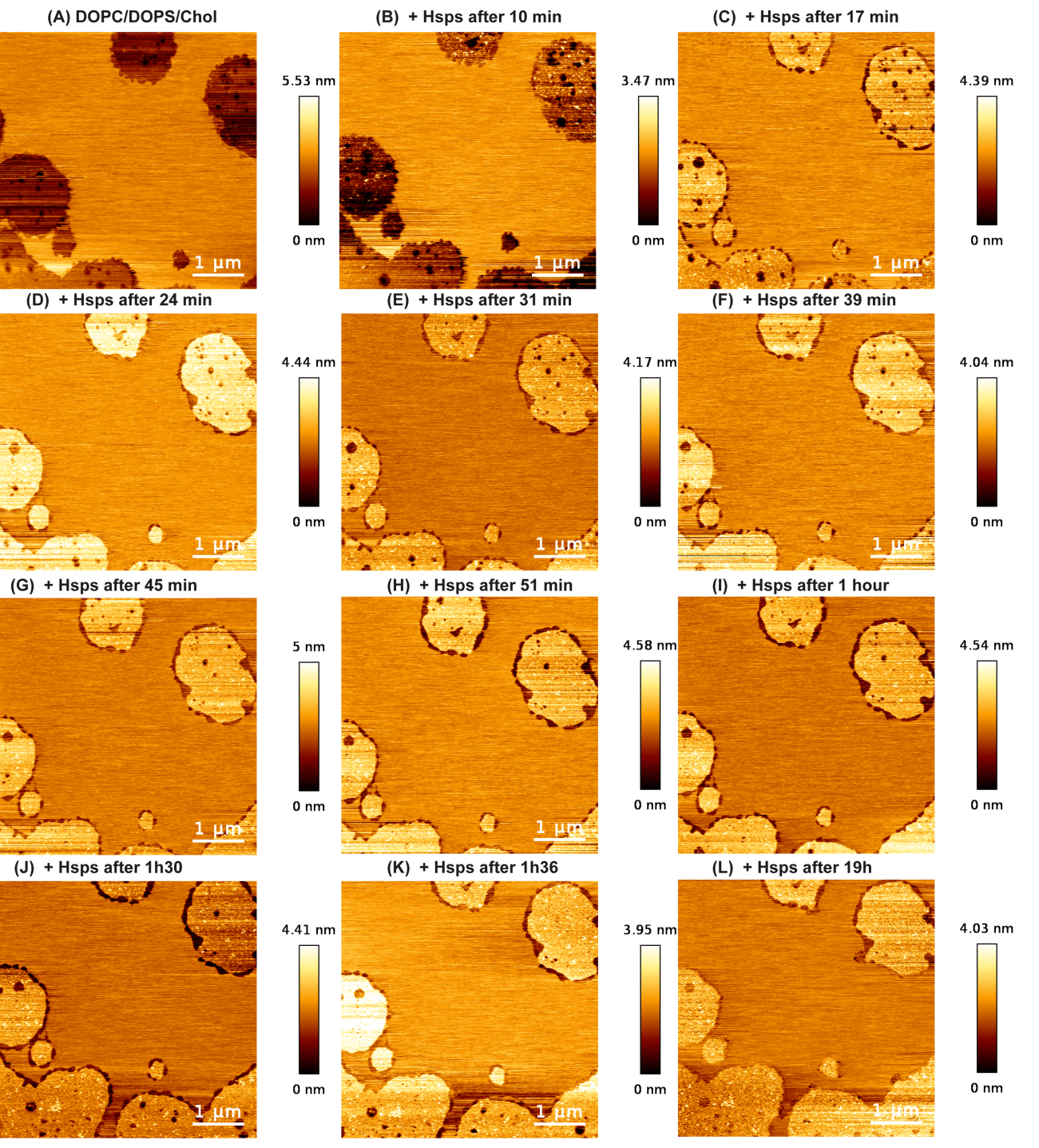
*

**Fig. S3:** AM-AFM topography images in HEPES buffer at room temperature, of DOPC/DOPs/Chol bilayer (50/20/30 mol%) deposited on mica substrate before addition of Hsp70 (A) and after different time of incubation with Hsp70 at a concentration of 100 µg/ml are shown in (B-L).

**Supplementary Figure S4: XRR curves of DOPC in the presence of Hsp70 proteins and their corresponding scattering length density profiles.**


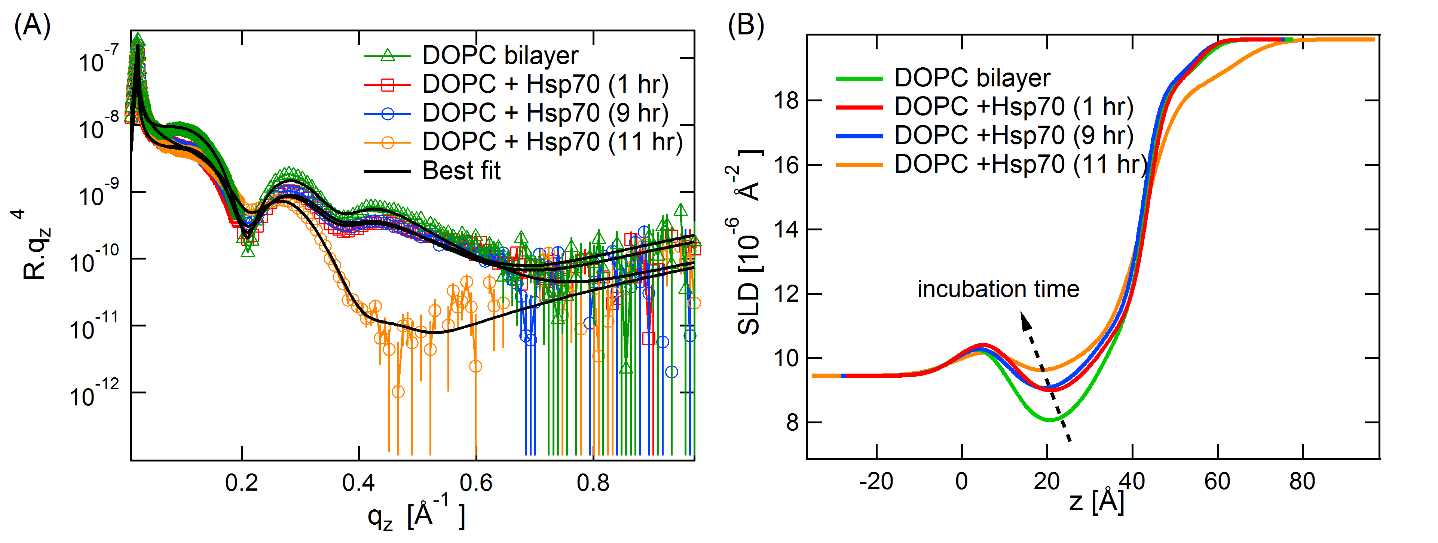
**Fig. S4**: (A) XRR curves of DOPC in the presence of Hsp70 proteins at different incubation times.(B) The reconstructed scattering length density profiles corresponding to XRR curves in A. Note that DOPC bilayer curve is measured independently and shown here for comparison.

**Figure S5: XRR curves of DOPC / DOPS in the presence of Hsp70 proteins at different incubation times.**


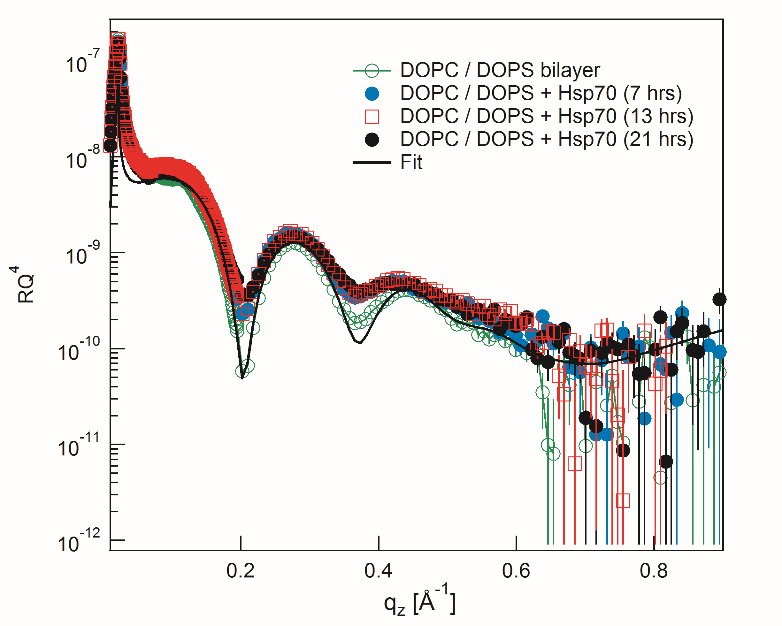


**Fig. S5**: XRR curves of DOPC / DOPS in the presence of Hsp70 proteins at different incubation times. The DOPC / DOPS bilayer curve is measured independently and shown here for comparison. Note that the DOPC / DOPS bilayer is intact in the presence of Hsp70 for more than 21 hours.

**Supplementary Table S1: The obtained structural parameters from XRR curves of DOPC in the presence of Hsp70 at different incubation times.**

**Table S1**

Structural parameters obtained from the best matching fits of XRR curves in Fig. S4.

|  | **d (Å)** | **ρ (e^-^ × Å^−3^ )** | **σ (Å)** |
| --- | --- | --- | --- |
|  |  | ***DOPC bilayer*** |  |
| **Outer head group** | 11 | 0.387 | 5.7 |
| **Hydrocarbonchains** | 24 | 0.277 | 5.4 |
| **Inner head group** | 8.6 | 0.455 | 5 |
|  | ***DOPC + Hsp70 (~ 1 h)*** | | |
| **Outer head group** | 12.1 | 0.394 | 4.9 |
| **Hydrocarbonchains** | 22.5 | 0.275 | 5.6 |
| **Inner head group** | 9.1 | 0.494 | 7.8 |
|  |  | ***DOPC + Hsp70 (~ 9 h)*** |  |
| **Outer head group** | 10.3 | 0.385 | 5.8 |
| **Hydrocarbonchains** | 23.8 | 0.314 | 5.3 |
| **Inner head group** | 8.6 | 0.447 | 8.3 |
|  |  | ***DOPC + Hsp70 (~ 11 h)*** |  |
| **Outer head group** | 12.1 | 0.372 | 7.6 |
| **Hydrocarbonchains** | 22 | 0.319 | 5.4 |
| **Inner head group** | 8.6 | 0.433 | 14.5 |

Note that the electron densities of the lipid layers are becoming closer to the measured electron density of the Hsp70 protein layer (*ρ_measured_* = 0.372 e^-^/Å^3^) which indicates the replacement of lipid bilayer with protein molecules.

**Supplementary Table S2: The obtained structural parameters from XRR curves of DOPC /DOPS.**

The structural parameters are presented here only for DOPC / DOPS bilayer since the change in the XRR curves of the bilayer bound to Hsp70 are minor changes.

**Table S2**

Structural parameters obtained from the best matching fits of XRR curve of DOPC / DOPS bilayer presented in Fig. S5.

|  | **d (Å)** | **ρ (e^-^ × Å^−3^ )** | **σ (Å)** |
| --- | --- | --- | --- |
|  |  | ***DOPC / DOPSbilayer*** |  |
| **Outer head group** | 10.4 | 0.400 | 4.1 |
| **Hydrocarbonchains** | 25.5 | 0.233 | 5.0 |
| **Inner head group** | 7.8 | 0.423 | 7 |
